# Vitronectin Destroyed Intestinal Epithelial Cell Differentiation through Activation of PDE4-Mediated Ferroptosis in Inflammatory Bowel Disease

**DOI:** 10.1155/2023/6623329

**Published:** 2023-07-19

**Authors:** Wenxu Pan, Li Xiang, Xinhua Liang, Wenjun Du, Junhong Zhao, Song Zhang, Xuan Zhou, Lanlan Geng, Sitang Gong, Wanfu Xu

**Affiliations:** ^1^The First Affiliated Hospital of Jinan University, Jinan University, Guangzhou, China; ^2^Department of Gastroenterology, Guangzhou Women and Children's Medical Center, Guangzhou Medical University, Guangzhou 510623, China; ^3^Department of Allergy, Immunology and Rheumatology, Guangzhou Women and Children's Medical Center, Guangzhou 510623, China; ^4^Department of Hematology, Zhujiang Hospital, Southern Medical University, Guangzhou, China

## Abstract

**Objective:**

Vitronectin (VTN) has been reported to trigger cell pyroptosis to aggravate inflammation in our previous study. However, the function of VTN in inflammatory bowel disease (IBD) remains to be addressed.

**Methods:**

Real-time PCR and western blotting were performed to analyze VTN-regulated intestinal epithelial cell (IEC) differentiation through ferroptosis, and immunofluorescence (IF), luciferase, and chromatin immunoprecipitation were used to identify whether VTN-modulated ferroptosis is dependent on phosphodiesterase 4 (PDE4)/protein kinase A (PKA)/cyclic adenosine monophosphate-response element-binding protein (CREB) cascade pathway. *In vivo* experiment in mice and a pilot study in patients with IBD were used to confirm inhibition of PDE4-alleviated IECs ferroptosis, leading to cell differentiation during mucosal healing.

**Results:**

Herein, we found that caudal-related homeobox transcription factor 2-mediated IECs differentiation was impaired in response to VTN, which was attributed to enhanced ferroptosis characterized by decreased glutathione peroxidase 4 (GPX4) and solute carrier family 7 member 11 expression. Inhibition of ferroptosis in IECs rescued the inhibitory effect of VTN on cell differentiation. Further analysis showed that VTN triggered phosphorylation of PDE4, leading to inhibit PKA/CREB activation and CREB nuclear translocation, which further reduced GPX4 transactivation. Endogenous PKA interacted with CREB, and this interaction was destroyed in response to VTN stimulation. What is more, overexpression of CREB in CaCO_2_ cells overcame the promotion of VTN on ferroptosis. Most importantly, inhibition of PDE4 by roflumilast or dipyridamole could alleviate dextran sulfate sodium-induced colitis in mice and in a pilot clinical study confirmed by IF.

**Conclusions:**

These findings demonstrated that highly expressed VTN disrupted IECs differentiation through PDE4-mediated ferroptosis in IBD, suggesting targeting PDE4 could be a promising therapeutic strategy for patients with IBD.

## 1. Introduction

Inflammatory bowel disease (IBD), including ulcerative colitis (UC) and Crohn's disease (CD), was chronic relapsing and life-threatening gastroenterological inflammatory disease characterized by impairment of intestinal epithelial cells (IECs) differentiation [1, 2]. The level of caudal-related homeobox transcription factor 2 (CDX2) expression has been confirmed to be critical for IECs differentiation during mucosal healing, which further induced mucin2 (MUC2), villin and tight junction protein-1 expression to maintain intestinal mucosal integrity [3–5]. Our previous study has demonstrated that the dynamic balance between the cyclic adenosine monophosphate (cAMP)-response element-binding protein (CREB)-CREB-binding protein (CBP) interaction and the nuclear factor *κ*B (NF-*κ*B)-CBP complex is critical for intestinal epithelial barrier and intestinal inflammation [1]. Inhibition of phosphodiesterase 4 (PDE4) by dipyridamole (DIP) could increase the abundance of CD8^+^CD39^+^ T cells in colonic mucosa, alleviating intestinal inflammation, which is attributed to the fact that PDE4 inhibition is well known to generate the intracellular level of cAMP, leading to trigger activation of protein kinase A (PKA)/CREB cascade pathway to induce the synthesis of CD39 [6]. Furthermore, a great deal of attention has been attracted by a serial of studies that inhibition of PDE4 could be a promising therapeutic strategy for IBD, including improvement of intestinal barrier function, intestinal fibrosis, inflammation, and the composition of gut microbiota [7–11]. However, the potential role of PDE4/cAMP/PKA signaling in IECs differentiation during mucosal healing in IBD remained to be identified.

Ferroptosis, a kind of iron-dependent regulatory necrosis, is characterized by the downregulation of the antioxidant peroxidase glutathione peroxidase 4 (GPX4), which regulated clearance of reactive oxygen species (ROS), followed by the accumulation of lipid peroxidation products or inhibition of the system Xc-, a cystineglutamate antiporter composed of solute carrier family 7 member 11 (SLC7A11) (system Xc-) that mediates the uptake of extracellular cystine, a major precursor for glutathione (GSH) biosynthesis [12]. Recently, accumulating studies have revealed that the pathogenesis mechanism of IBD was associated with ferroptosis activation [13–16]. For instance, ferrostatin-1, a well-known small molecule that specifically inhibits ferroptosis, ameliorated TNBS-induced colitis through increasing GPX4 expression and reducing tumor necrosis factor-*α*, IL-1*β*, and IL-6 mRNA levels [17], and *in vitro* model of the intestinal epithelium cell differentiation further showed that GPX4 expression level was increased during a time course of enterocytic cell differentiation [18], interestingly, phosphorylation of CREB was uncovered to initiate GPX4 gene transcription, including IL-10 and GPX4 [19, 20], suggesting CREB signaling-mediated ferroptosis is closely associated with IEC differentiation during mucosal healing. However, the regulation of ferroptosis in IBD during mucosal healing remained to be elucidated.

Vitronectin (VTN), a multifunctional glycoprotein, has been shown to be enriched in the serum, extracellular matrix, and platelets, involving in such as the regulation of blood coagulation pathways, and the formation of membrane attack complex (MAC), cell adhesion and migration, and tissue remodeling through *α*v*β*3 and *α*v*β*5 integrin signaling [21, 22]. Intestinal fibrosis was characterized by an excessive accumulation of extracellular matrix (ECM) proteins at the injured sites, such as collagen, FN, and VTN [23]. The ECM interacted with cells to regulate diverse functions, including proliferation, migration, and differentiation [24, 25]. For instance, VTN promoted the specification of hematopoietic-fated mesoderm and enhanced HE generation from mesodermal progenitor cells via *α*v*β*3 and *α*v*β*5 integrins [26]. However, the function of VTN in IBD characterized by mucosal injury remained to be explored. In recent, our work has revealed that VTN could trigger NLR family, pyrin domain containing 3 (NLRP3) inflammasome activation through NF-*κ*B, leading to cell pyroptosis in THP-1-derveried macrophages. Herein, we further demonstrated that the VTN inhibited IEC differentiation as noted by the decreased differentiation markers MUC2 and CDX2, which was attributed to activation of ferroptosis featured by enhanced GPX4 and SLC7A11 expression. Inhibition of ferroptosis largely rescued the effect of VTN on CDX2-mediated IECs differentiation. Mechanically, VTN treatment led to a significant activation of PDE4B/C/D, which further decreased phosphorylation of PKA/CREB, leading to inhibit nuclear translocation of CREB and GPX4 transactivation. Endogenous PKA interacted with CREB, and this interaction was dramatically disrupted in response to VTN treatment. What is more, roflumilast (Rofi), an inhibitor of PDE4, could reverse the effect of VTN. Most importantly, inhibition of PDE4 by DIP or Rofi could alleviate dextran sulfate sodium (DSS)-induced colitis and IBD. Collectively, these findings provided the mechanism through which VTN-regulated IBD and targeting PDE4 could be an effective therapeutic approach to improve IBD.

## 2. Materials and Methods

### 2.1. Chemical Reagents and Antibodies

VTN (459 a.a, HEK293, His), RSL3 (HY-100218A), Ferrostatin-1 (HY-100579), DIP (HY-B0312), and Rofi (HY-15455S2) were purchased from MedChemExpress (Shanghai, China). Antibodies targeting GPX4 (67763-1-Ig, 1 : 200 for immunofluorescence (IF), 1 : 2,000 for western blotting (WB)), SLC7A11 (26864-1-AP, 1 : 200 for IF, 1 : 2,000 for WB), CDX2 (60243-1-Ig, 1 : 200 for IF, 1 : 2,000 for WB), CREB1 (12208-1-AP, 1 : 2,000 for WB), PRKACA (27398-1-AP, 1 : 2,000 for WB), *α*-tubulin (66031-1-Ig, 1 : 2,000 for WB) were from Proteintech Company (Wuhan, China). Phospho-CREB1 (Ser133) (YP0075, 1 : 2,000 for WB), Phospho-PKA (Thr198) (YP0226, 1 : 2,000 for WB), Phospho-PDE4 (YP0668, 1 : 100 for IF, 1 : 2,000 for WB), EpCAM (YM0219, 1 : 200 for IF), EpCAM (YM6053, 1 : 200 for IF) were from Immunoway Research (Plano, USA). Antibodies against MUC2 (ab272692, 1 : 2,000 for WB) and PDE4 (ab99409, 1 : 2,000 for WB) were from Abcam (Cambridge, UK). Lamin A/B (sc-376248, 1 : 3,000 for WB) was from Santa Cruz Biotechnology (Dallas, Texas, USA). All unconjugated secondary antibodies were from Beijing Ray Antibody Biotech (Beijing, China). All ultrapure reagents were from Promega (Madison, WI, USA).GPX4 plasmid was purchased from Youbio (Hunan, China), siRNA was synthesized by Genepharma (Shanghai, China).

### 2.2. Cell Culture, Treatment, and Transfection

CaCO_2_ and HT-29 cells lines were purchased from the American Type Culture Collection (ATCC, Manassas, USA) and cultured in Dulbecco's Modified Eagle Medium (DMEM) containing 10% fetal bovine serum (FBS), 100 U/ml penicillin and 100 mg/ml streptomycin. DMEM and FBS were purchased from Gibco (Thermofisher Scientific, USA). Cells were maintained in a humidified incubator at 37°C and 5% CO_2_. For treatment, the cells were treated with VTN at a final concentration of 5 *μ*g/ml for 48 hr. For transfection, the plasmids, or siRNAs targeted PDE4 were delivered into cells with lipofectamine 3000. The following siRNA sequences were used: PDE4A, 5ʹ-CAGGAGUCGUUGGAAGUUA-3ʹ; PDE4B, 5ʹ-UUAGAAGCCAUCUCACUGACAGACC-3ʹ; PDE4D, 5ʹ-GAGUUCUUCUUCUUGAUAA-3ʹ [27].

### 2.3. Real-Time PCR Analysis

As described in our previous work [28, 29], briefly, after treatment for 24 hr, Total RNA was extracted and reverse transcripted into cDNA according to Beyozol RNA Isolation Kit and the All-in-one™ first-strand cDNA synthesis kit (GenecopoeiaTM, FulenGen), respectively. Quantitative PCR (qPCR) was carried out using the All-in-one™ qPCR mix (GenecopoeiaTM, FulenGen) according to the manufacturer's instructions. Primer used in this study as followed: GPX4 forward, 5′-GAGGCAAGACCGAAGTAAACTAC-3′; reverse, 5′-CCGAACTGGTTACACGGGAA-3′; SLC7A11 forward, 5′-ACGGTGGTGTGTTTGCTGTCTC-3′; reverse, 5′-GCTGGTAGAGGAGTGTGCTTGC-3′; CDX2 forward, 5′-CTCGGCAGCCAAGTGAAAACCA-3′; reverse, 5′-GCTTTCCTCCGGATGGTGATGTA-3′; MUC2 forward, 5′-GAGGGCAGAACCCGAAACC-3′; reverse, 5′-GGCGAAGTTGTAGTCGCAGAG-3; GAPDH, forward, 5′-AACGGATTTGGTCGTATTGGG-3′, reverse, 5′-CCTGGAAGATGGTGATGGGAT-3′.

### 2.4. Protein Extraction and Western Blotting

Total proteins from cells were extracted with RIPA lysis buffer (Biosharp, China). Nuclear and cytosolic proteins were separated by nuclear extraction kit (Thermofisher Scientific, USA) according to the manufacturer's instructions. Protein concentration was determined using BCA Protein Assay Kit (Thermofisher Scientific, USA). WB was performed in a previous study [30]. Briefly, proteins were subjected from SDS-PAGE and transferred into nitrocellulose transfer membrane to incubate with 5% slim milk in PBS/0.05% Tween for 1 hr. The primary antibodies were added and incubated overnight at 4°C, followed by incubation with secondary antibodies (Jackson ImmunoResearch, UK) for 1 hr at room temperature. Proteins were imaged using an enhanced chemiluminescence (Perkin Elmer).

### 2.5. ROS Detection

DCFH-DA staining was used to detect the ROS level. Briefly, after digestion, the cells were reseeded at a density of 10^5^/ml for 24 hr to allow to adhere. HT-29 or CaCO_2_ cells were treated with or without VTN for 48 hr, washed with PBS and incubated with DCFH-DA solution for another 20 min at 37°C and 5% CO_2_. The ROS level was visualized and captured under fluorescence microscopy.

### 2.6. Immunofluorescence

IF was performed as previously described [31]. For IECs, The CaCO_2_ cells were digested and reseeded at a density of 0.5 × 10^5^/ml in 6-well plates overnight. After VTN treatment, cells were fixed in 4% paraformaldehyde for 15 min, permeabilized in 0.5% Triton X-100 for 20 min and then blocked in 10% goat serum for 30 min. Cells were incubated with the primary antibodies overnight at 4°C. For tissue slides were deparaffinized, incubated with blocking buffer (PBS with 5% normal donkey or goat serum and 0.3% Triton X-100) at room temperature for 1 hr, and stained with primary antibodies overnight in a wet chamber at 4°C in the dark and then incubation was required for another 1 hr with secondary antibodies (Jackson ImmunoResearch, UK) at room temperature. The coverslips were mounted onto glass slides with prolonged gold reagent after staining the nuclei with 4′,6-diamidino-2-phenylindole (DAPI). Stained cells were visualized using a laser-scanning confocal fluorescent microscope.

### 2.7. Immunoprecipitation (IP)

As described in our previous study [29], after treatment with or without VTN for 1 hr, the total cells were harvested and lysated with RIPA for 15 min. lysis were incubated with anti-PKA or nonspecific immunoglobulin (IgG) overnight at 4°C. The beads were washed with ice-cold RIPA buffer and incubated with lysate for another 2 hr. Elution was performed by adding 2x SDS-PAGE protein sample buffer and boiling at 95°C for 10 min. Protein expression was assayed in western blots.

### 2.8. Luciferase Assay

As described in our previous work [32], luciferase reporter plasmid and internal control plasmid (pGL4.74) were co-transfected into cells using lipofectamine 3000 according to manufacturer's instruction. Twenty-four hours after transfection, the cell was treated with or without VTN for another 24 hr, and firefly and renilla luciferase value were measured using the Dual-Luciferase Reporter Assay System (Promega).

### 2.9. Chromatin IP

As described in our previous study [28, 33], chromatin immunoprecipitation (ChIP) assay was performed according to the protocol of Sample ChIP(R) Plus Kit (Magnetic Bead) (Cell Signal Technology, 9005) with anti-CREB or negative control anti-IgG. The precipitated DNAs were analyzed and quantified by using real-time PCR analysis with the following primers [20]: GPX4 forward: 5′- AAGCGAGCATGCGCAGTCGCCAA-3′; reverse: 5′-GGACGCGCGTCGGCTTTCCGCG-3′.

### 2.10. Crypt Isolation, Culture, and the Generation of Organoids

Briefly, the intestine was collected from 8-week mice and opened longitudinally to wash with cold PBS to remove contents, subsequently followed by cutting into 2–4 cm pieces, which were further digested using Gentle Cell Dissociation Reagent cGMP (STEMCELL, 100-0485) on a shaker with a speed of 300 rpm at 4°C to release crypts. After filtration with 70 *μ*m strainer and centrifugation, the crypts were collected, washed with DPBS, and suspended with intestiCult™ Organoid Growth Medium (Mouse, STEMCELL, 06005) to culture. 5 *μ*g/ml VTN was added to observe the effect of VTN on intestinal organoids generation. After capture, intestinal organoids were rinsed three times in ice-cold PBS, fixed with 4% paraformaldehyde, and embedded in paraffin, cut into 3 *μ*m sections. The sections were deparaffinized and prepared for IF.

### 2.11. A Pilot Clinical Study

Based on the Helsinki Declaration, the work is approved by the Medical Ethics Committee of Guangzhou Women and Children's Medical Center (ID:2017021504) and clinical trial (ID:2017052401). Informed written consent was obtained from the legal guardians of all participants. Patients were recruited in the Women and Children's Medical Center received DIP study. The intestinal mucosa tissue was collected from children with IBD before and after treatment, respectively, after we got informed consent from the patients in the Department of Gastroenterology, which was approved by the Medical Ethical Review Board, named Scientific Research Committee of Guangzhou Women and Children's Medical Center.

### 2.12. DSS-Induced Chronic Colitis Model

Mice aged from 6–8 weeks were fed with drinking water containing with 2% DSS (36,000–50,000 MW, MP Biomedicals) and treated with DIP (10 mg/kg body weight, Sigma–Aldrich) or vehicle (2% DMSO, 10% ethanol, 88% corn oil) twice per day for 2 months. Control mice were given regular food and water. Mice were monitored daily and euthanized if they reached more than 20% body weight loss.

### 2.13. Statistical Analysis

All statistical analyses were performed using Prism 9. Each experiment was performed for three biological replicates. One-Sample *t* test was used to analyze the difference in qPCR assay; the band intensity was quantified by *t* test. The luciferase activity was analyzed by two-ANOVA with multiple comparisons, followed by Bonferroni post hoc test for significance. All statistical analyses utilized a 0.05 level of significance.

## 3. Results

### 3.1. VTN-Induced Dysregulation of Intestinal Cell Differentiation

**Figure 1:**
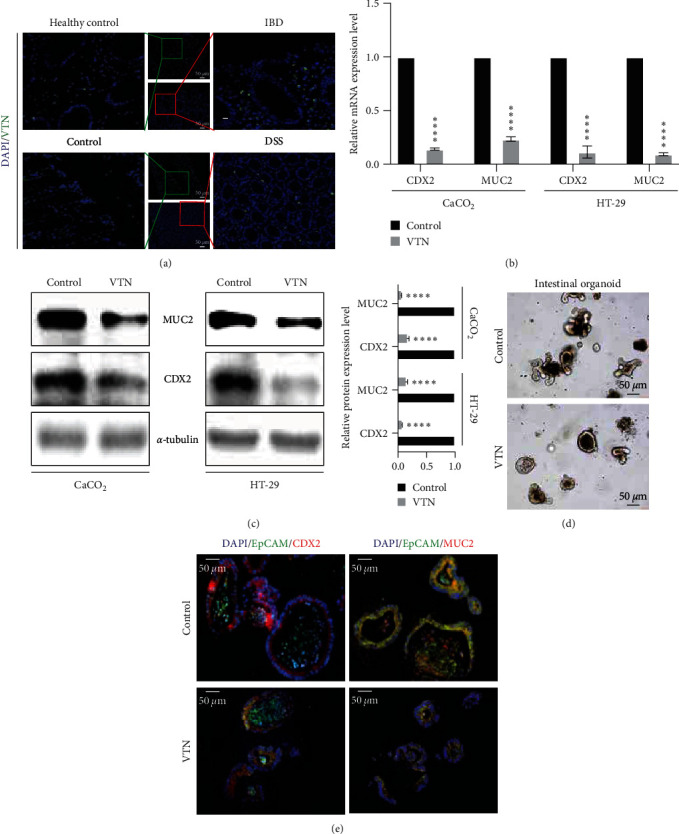
VTN suppressed intestinal epithelial cell differentiation. (a) Immunofluorescence of VTN expression level in indicated intestinal tissue from clinical sample and colitis model. (b) Real-time PCR and (c) Western blotting and quantified analysis were performed to detect the effect of VTN on CDX2 and MUC2 expression. Data presented as the mean ± s.e.m. of three independent experiments and were analyzed by *t* test,  ^*∗∗∗∗*^*p* < 0.0001. (d) Intestinal organoid assay was used to explore the effect of VTN on intestinal organoid generation. (e) The immunofluorescence was performed to analyze CDX2 and MUC2 expression in intestinal organoid in response to VTN stimulation described as (d).

To explore the possible role of VTN in IBD, a set of intestinal tissue from IBD and healthy control and DSS-induced colitis were collected to detect the VTN expression level through IF. The results showed that, in comparison with control group, VTN was significantly accumulated in extracellular region of epithelial cell labeled by EpCAM in IBD and DSS-induced colitis (Figure 1(a)). To further address the possible role of VTN in enterocytes differentiation, CaCO_2_ and HT-29 cells have served as useful *in vitro* model to delineate the effect of VTN in IEC differentiation, which was attributed to the fact that CaCO_2_ cells can differentiate into an enterocyte-like phenotype spontaneously after overconfluence [4, 34, 35]. The result from qPCR showed that VTN treatment in CaCO_2_ and HT-29 cells led to significant downregulation of CDX2 and MUC2 at the mRNA level compared with control group (Figure 1(b)). We further investigated changes in the expression of these targets at the protein level by WB. Protein levels of CDX2 and MUC2 were largely blocked in HT-29 and CaCO_2_ cells after stimulation with VTN (Figure 1(c)). In line with this, the results from intestinal organoid showed that intestinal organoid was impaired in response to VTN treatment, which was confirmed by decreased CDX2 expression from IF (Figures 1(d) and1(e)). These findings suggested VTN has a potent impairment role in enterocyte differentiation.

### 3.2. VTN Destroyed Intestinal Epithelial Barrier is Dependent on Ferroptosis

**Figure 2:**
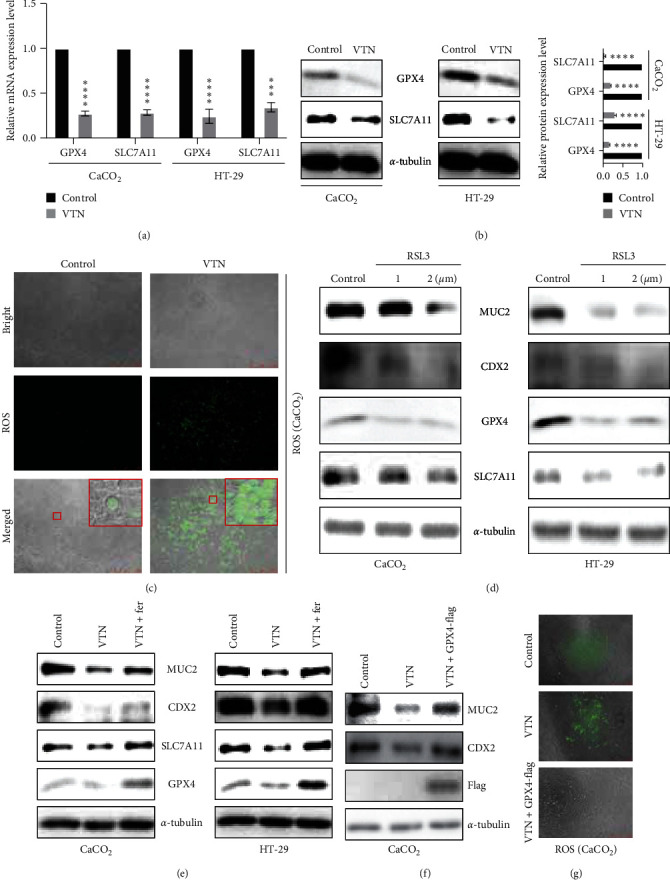
VTN promoted ferroptosis to suppress cell differentiation. (a) Intestinal epithelial cells were digested and reseeded in a 6-well plate overnight; after 12-serum-free treatment, the cells were treated with or without VTN (5 *μ*g/ml) for another 48 hr, real-time PCR were performed to analyze *GPX4* and *SLC7A11* expression at mRNA level, data presented as the mean ± s.e.m. of three independent experiments and were analyzed by *t* test,  ^*∗∗∗∗*^*p* < 0.0001. (b) CaCO_2_ and HT-29 cells were treated and described as (a). The total protein was collected and subjected by western blotting to detect *GPX4* and *SLC7A11* expression at protein level; the band intensity was measured and quantified by *t* test, data presented as the mean ± s.e.m. of three independent experiments and were analyzed by *t* test,  ^*∗∗∗*^*p* < 0.001,  ^*∗∗∗∗*^*p* < 0.0001. (c) CaCO_2_ cells were treated described as (a), and the ROS level was detected according to the manufacturer's instruction. (d) After starvation, CaCO_2_ and HT-29 cells were treated as indicated for 48 hr, and the total protein level was collected; WB was employed to detected activation of ferroptosis by RSL3 on intestinal epithelial cell differentiation. (e) After serum starvation for 24 hr, CaCO_2_ and HT-29 cells were treated with or without VTN, followed by ferroptosis inhibitor ferrostatin-1 (Fer) (10 *μ*M) stimulation for further 48 hr. The total lysates were collected to analyze indicated protein levels. (f) 24 hr after transfection with *GPX4* plasmid, CaCO_2_ cells were treated with or without VTN for another 24 hr to collect the total protein; WB was performed to detect indicated proteins. (g) CaCO_2_ cells were treated as (f), and ROS staining was used to analyze the ROS level.

Ferroptosis has been reported to play an important role in gut crypt-derived organoids [36], which focused us to confirm the possibility that the impaired intestinal cell differentiation caused by VTN treatment was attributed to the enhancement of ferroptosis. As expected, enhanced ferroptosis characterized by increased ROS level and decreased GPX4 and SLC7A11 expression at mRNA and protein levels was observed in HT-29 and CaCO_2_ cells in response to VTN stimulation (Figure 2(a)–2(c), Figure S1).

Next, RSL3, an inhibitor of GPX4, was used to detect the ferroptosis on IEC differentiation. As shown in Figure 2(d), the result showed that the protein level of CDX2 and MUC2 expression was significantly decreased in HT-29 and CaCO_2_ cells with ferroptosis activation after RSL3 treatment with 1 and 2 *μ*M, respectively. Most importantly, VTN treatment in HT-29 and CaCO_2_ cells led to impaired intestinal cell differentiation, which is overcome by Fer-1 stimulation (Figure 2(e)); in line with this, overexpression of GPX4 in CaCO_2_ cells could reverse the inhibitory effect of VTN on CDX2, MUC2 expression and ROS level (Figures 2(f) and 2(g)). Taken together, these findings suggested VTN disrupted intestinal cell differentiation is dependent on GPX4-mediated ferroptosis.

### 3.3. CREB was Required for VTN-Induced Ferroptosis

**Figure 3:**
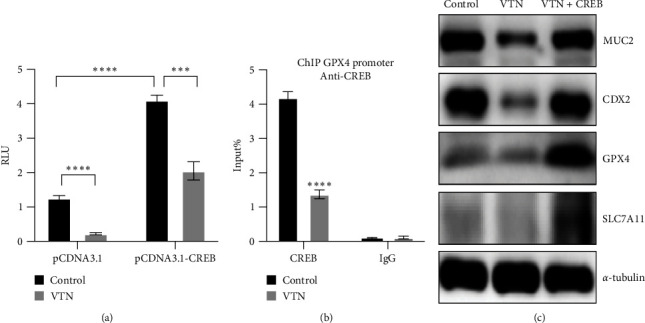
VTN regulated ferroptosis through CREB. (a) Internal control pGL4.17 and *GPX4* promoter reporter plasmid together with pCDNA 3.0 or CREB plasmid were transfected into HT-29 cells, followed by treated with or without VTN for 48 hr, relative luciferase activities unit were measured and analyzed by two-way analysis of variance (ANOVA) and Dunnett's multiple comparison test,  ^*∗∗∗*^*p* < 0.001,  ^*∗∗∗∗*^*p* < 0.0001, *n* = 3, data presented as the mean ± s.e.m. (b) ChIP analysis of binding of CREB protein to *GPX4* gene promoter in CaCO_2_ cells treated as indicated. Student's *t*-test, data presented as the mean ± s.e.m, relative luciferase activities unit were measured and analyzed by two-way analysis of variance (ANOVA) and Dunnett's multiple comparison test,  ^*∗∗∗*^*p* < 0.001,  ^*∗∗∗∗*^*p* < 0.0001, *n* = 3. (c) HT-29 cells were transfected with or without CREB for 24 hr, further incubation with VTN was performed for another 24 hr, the total lysate was collected and subjected from WB to examine indicated protein.

The above results have suggested that VTN-mediated ferroptosis played an important role in IEC differentiation. CREB has been confirmed to be involved in the transactivation of GPX4 [18, 20, 37] to further identify VTN-regulated GPX4-mediated ferroptosis that could be attributed to CREB. As shown in Figure 3(a), a luciferase reporter containing GPX4 promoter (GPX4-luc) was established and codelivered into HT-29 with internal control (pGL4.74), followed by treated with or without VTN for 48 hr. The results showed that ectopic expression of CREB largely increased, while VTN stimulation decreased GPX4 promoter activity in HT-29 cells. What is more, overexpression of CREB in HT-29 cells reversed the abolished GPX4 luminescence caused by VTN treatment. What is more, ChIP combined with qPCR analysis revealed that VTN treatment largely abolished the binding ability of CREB to *GPX4* gene promoter in HT-29 cells (Figure 3(b)). and overexpression of CREB in HT-29 overcame the inhibitory effect of VTN on GPX4 expression (Figure 3(c)). These results suggested that CREB was essential for VTN-mediated GPX4 transactivation.

### 3.4. VTN-Modulated PDE4/PKA/CREB Cascade Signaling

**Figure 4:**
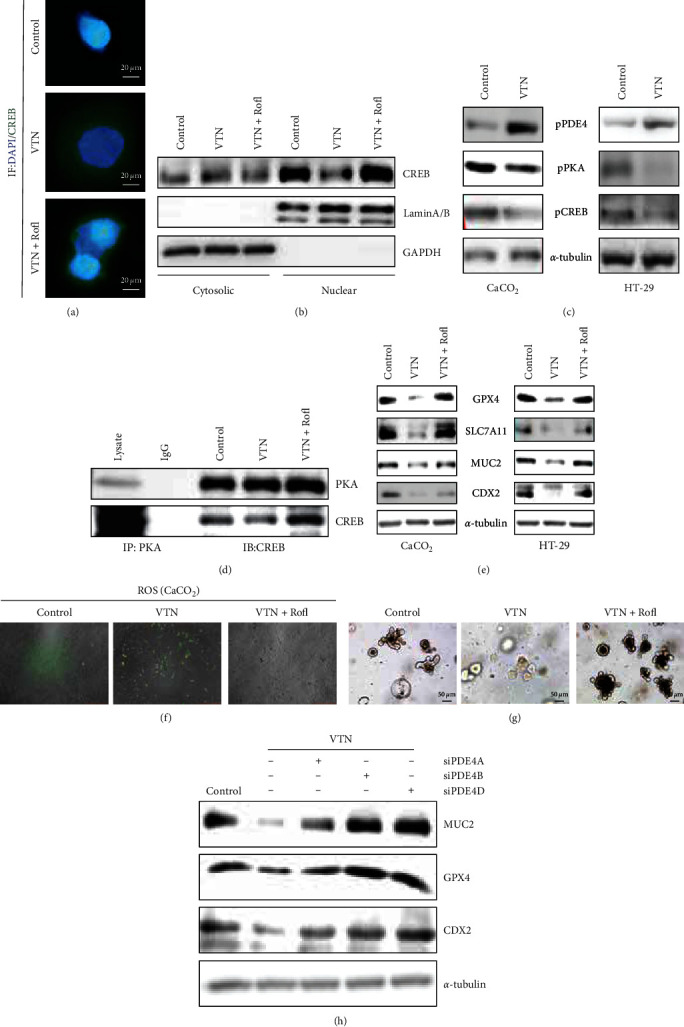
VTN modulated PDE4/PKA/CREB signaling. (a) Immunofluorescence was employed to display the CREB nuclear localization in CaCO_2_ cells treated with VTN or VTN combined with roflumilast (Rofl) for 1 hr. (b) CaCO_2_ cells were serum starved for 24 hr and then stimulated as indicated for an additional 1 hr. Nuclear and cytosolic CREB levels were determined by western blotting. GAPDH and lamin A/B were served as internal controls for the cytosolic and nuclear fractions, respectively. (c) After serum starved for 24 hr, CaCO_2_ and HT-29 cells were treated with or without VTN for 1 hr, the total proteins were collected and separated to detect indicated protein by western blotting. *α*-Tubulin was taken as internal control. (d) HT-29 cells were serum starved for 24 hr after 80% confluence, then stimulated as indicated for 1 hr. Immunoprecipitated (IP) was employed to analyze the interaction between PKA and CREB in response to VTN and VTN combined with Rofl. (e) After serum starved for 24 hr, the total cell was pretreated with Rofl for 1 hr; subsequently, followed by stimulation with or without VTN for further 48 hr, the total protein was collected to detect indicated protein. *α*-tubulin was served as internal control. (f) CaCO_2_ cells were treated with basic medium for 24 hr, followed by stimulation with VTN and VTN combined with Rofl for further 48 hr, the level of ROS was detected. (g) Intestinal organoids assay was performed to analyze the effect of VTN-Rofl on cell differentiation. (h) After transfection with indicated siRNA for 24 hr, HT-29 cells were starved for 12 hr and treated with or without VTN for another 24 hr; the total protein was collected to detect indicated proteins.

Activation of CREB is critical for its nuclear translocation to activate CREB-mediated target genes expression [38, 39], which focused us to explore the extract mechanism that VTN regulated CREB-mediated GPX4 expression using a subcellular fractionation analysis and immunofluorescent staining. As shown in Figure 4(a)–4(c), the nuclear location of CREB was drastically inhibited in CaCO_2_ cells in response to VTN treatment, which was attributed to phosphorylation of PDE4 triggered by VTN, leading to decrease phosphorylation of PKA/CREB. What is more, inhibition of PDE4 by Rofi (20 *μ*M) could reverse the effect of VTN on CREB localization. Most importantly, endogenous PKA interacted with CREB, and this interaction was disrupted by VTN (Figure 4(d)). In addition, inhibition of PDE4 activation by Rofi alleviated the inhibitory effect of VTN on GPX4 and SLC7A11 expression, reducing ROS production and ferroptosis, which further promoting CDX2 expression (Figures 4(e) and 4(f), Figure S1). In line with the above, the results from the intestinal organoid model also have confirmed that intestinal organoid was induced in present with VTN after Rofi stimulation (Figure 4(g)). To further confirm which PDE4 subtypes (PDE4A, B, C, and D) are involved in VTN-regulated ferroptosis and differentiation in IECs, we knockdown PDE4A, PDE4B, and PDE4D expression in HT-29 cells by siRNA transfection could reverse the effect of VTN on CDX2, MUC2, and GPX4 expression; the knockdown efficiencies were tested in Figure S1, while PDE4C level was minimal or absent (data not shown) (Figure 4(h)). Taken together, these results suggested that VTN-modulated PDE4/PKA/CREB cascade pathway to trigger ferroptosis, leading to destroy cell differentiation.

### 3.5. Inhibition of PDE4 Decreased Ferroptosis to Alleviate Colitis In Vivo

**Figure 5:**
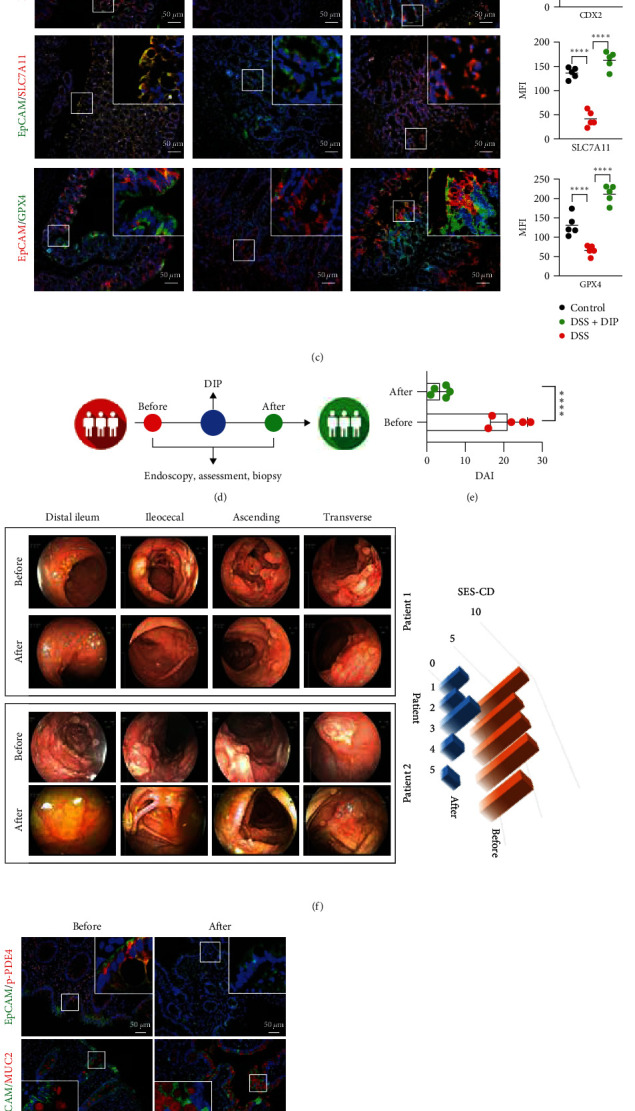
Inhibition of PDE4 alleviated colitis. In a DSS-induced acute colitis model, mice were treated with dipyridamole (10 mg/kg) daily for 1 month, and the body weight was recorded every 2 days (a) The statistical difference was performed by one-way ANOVA,  ^*∗∗∗∗*^*p* < 0.0001, control, *n* = 6; DSS + vehicle, *n* = 6; DSS + dipyridamole, *n* = 6, data presented as the mean ± s.e.m. (b) The representative image of colon length was captured and the statical analysis were performed by one-way ANOVA,  ^*∗∗∗∗*^*p* < 0.0001, data are represented as mean ± s.e.m.;  ^*∗∗∗∗*^*p* < 0.0001. (c) Immunofluorescence of indicated protein expression level was used to show the effect of DIP in DSS-induced colitis, quantitation was performed by Image J, and data presented as the mean ± s.e.m and analyzed by one-way ANOVA,  ^*∗∗∗∗*^*p* < 0.0001, MFI: mean fluorescence intensity. (d) Schematic view of IBD patients enrolled in a pilot clinical study received endoscopy assessment and biopsy collection. The information for the pilot study is provided in Table 1. (e) Analysis of clinical disease activity index (DAI) evaluation in five IBD children in indicated timepoint. Data are represented as mean ± s.e.m; *p* values were calculated by *t* test. *n* = 5,  ^*∗∗∗∗*^*p* < 0.0001. (f) The representative endoscopic images of before and after DIP administration in five children with IBD, and score for endoscopy was evaluated. (g) Immunofluorescence of indicated protein expression level was used to show the effect of DIP in patients with IBD, quantitation was performed by Image J, and data presented as the mean ± s.e.m and analyzed by paired *t* test,  ^*∗∗∗∗*^*p* < 0.0001, MFI: mean fluorescence intensity. (h) Schematic model of VTN-regulated intestinal epithelial cell differentiation through ferroptosis.

The above results suggested VTN destroyed IEC differentiation through PDE4-mediated ferroptosis; next, we sought to determine whether the clinically approved PDE4 inhibitor DIP could alleviate ferroptosis, contributing to cell differentiation. As shown in Figures 5(a) and 5(b), in a DSS-induced acute colitis model, bodyweight and colon length were significantly increased in response to DIP treatment in comparison with mice received DSS treatment alone. Further analysis showed ferroptosis was significantly inhibited, characterized by enhanced GPX4 and SLC7A11 expression, contributing to IEC differentiation featured by enhanced CDX2 and MUC2 expression (Figure 5(c)). What is more, a pilot clinical study in five IBD patients confirmed with endoscopy was performed to evaluate the effect of DIP on mucosal healing in clinic. The detailed information of subjects enrolled in this work is listed in Table 1. DIP administration largely improved the mucosal healing confirmed by endoscopic examination, and scores for SES-CD and disease activity index (DAI) were significantly improved after the treatment (Figure 5(d)–5(f)). The immunofluorescent staining has confirmed that DIP treatment could promote IEC differentiation characterized by increased CDX2 and MUC2 expression, while a remarkable reduction of ferroptosis was observed featured by enhanced GPX4 and SLC7A11 expression (Figure 5(g)). Collectively, our findings suggested that inhibition of PDE4B could boost mucosal healing by modulating ferroptosis.

## 4. Discussion

Up to now, there is no available study about the function of VTN in IEC differentiation during mucosal healing in IBD. In this work, as shown in Figure 5(h), we rigorously demonstrated that VTN derived a novel signaling pathway in modulating intestinal cell differentiation in IBD through PDE4-mediated ferroptosis. Highly expressed VTN in IECs led to destroy intestinal cell differentiation is dependent on ferroptosis. Mechanistically, VTN triggered PDE4 activity, leading to degrade cAMP and inhibit phosphorylation of PKA/CREB to decrease GPX4 transactivation to initiate ferroptosis, which in turn abolished CDX2 expression and destroyed IEC differentiation. What is more, endogenous PKA interacted with CREB, and this interaction was disrupted in response to VTN stimulation in IEC. Interestingly, inhibition of PDE4 by Rofi could counteract the effect of VTN on the complex of PKA-CREB. Most importantly, inhibition of PDE4 by Rofi could rescue the symptoms of DSS-induced colitis *in vivo*, and DIP administration could alleviate clinical symptoms of IBD in a clinical pilot study. Taken together, these results provided evidence to show the novel role of VTN in IBD and supported targeting PDE4 as a promising approach for patients with IBD.

The previous work has demonstrated that VTN, the ligand of *α*V*β*3 integrin, stimulated proliferation and suppressed apoptosis in intestinal smooth muscle cell hyperplasia in stricturing CD [40]. Our lab showed that VTN triggered NLRP3 inflammasomes activation, leading to trigger pyroptosis to aggravate inflammation in THP-1-derived macrophages [41], and in the current study, we further showed the potent pro-IBD effect of VTN originated in its ability to decrease SLC7A11 and GPX4 expression to induce ferroptosis, which leading to inhibit CDX2-mediated cell differentiation during mucosal healing. In fact, the results from Figure 5(h), both GPX4 and SLC7A11 expression in IECs labeled with EpCAM were enhanced along the intestinal crypt-villus axis characterized by cell differentiation. In line with this, inhibition of ferroptosis by ferrostatin-1 could rescue the inhibitory effect of VTN on cell differentiation, suggesting the VTN-regulated IECs differentiation is dependent on ferroptosis. However, further work was required to address the detailed mechanism through which VTN-mediated ferroptosis to regulate IECs differentiation, even intestinal barrier function. In addition to ferroptosis, whether cuproptosis involved in VTN-mediated impaired cell differentiation in IBD remained to be confirmed in future work.

cAMP is the classic second messager, which could activate PKA/CREB cascade pathway or be hydrolyzed into AMP by PDE4 [42]. Recently, the serial work suggested PDE4 could be a promising therapeutic target for various diseases, such as nephrotic syndrome [43], acute myeloid leukemia [44], idiopathic pulmonary fibrosis [45], heart failure [46], and colitis [8]. Interestingly, phosphorylation of PDE4 is a critical event in VTN-induced ferroptosis and dysfunction of cell differentiation, as confirmed by the results that inhibition of PDE4 by DIP could alleviate the effect of VTN on ferroptosis-dependent cell differentiation in IECs, and DIP administration could drastically improve CDX2-mediated cell differentiation in an animal model and in a pilot study. What is more, further analysis showed that PDE4B and PDE4D played a critical role in VTN-mediated ferroptosis. In addition to ferroptosis and cell differentiation, further investigation was needed to explore the other function of VTN in IBD in the future, including intestinal mucosal immunity, drug resistance, and intestinal fibrosis, and whether VTN regulated the phosphorylation of PDE4 in an integrin-dependent manner could be confirmed in next work, although the studies have confirmed that intergern was activated by VTN stimulation [26, 47, 48]. However, further studies are required to elucidate the mechanism regulating the VTN expression under physiological or pathological conditions, even whether there is a positive feedback loop between VTN-ferroptosis.

In summary, our data have established a novel mechanism through which VTN triggered PDE4-mediated ferroptosis to destroy IEC differentiation and inhibition of PDE4 phosphorylation could be a promising therapeutic strategy to improve intestinal mucosal healing in IBD.

## Figures and Tables

**Table 1:** Detailed information of patients enrolled in a clinical pilot study.

Patient ID	Age	Gender	Type of IBD	Therapeutic regimen	DAI	
					Before	After
1	12	F	CD	Enteral nutrition, DIP	27	5
2	15	M	CD	Enteral nutrition, DIP	25	6
3	12	M	CD	Anti-TNF, DIP	17	5
4	12	M	CD	Anti-TNF, DIP	16	1
5	7	M	CD	Anti-TNF, DIP	14	5

## Data Availability

The datasets generated during and/or analyses during the current study are available from the corresponding author upon reasonable request.

## Abbreviations

CDX2: Caudal-related homeobox transcription factor 2
CD: Crohn's disease
ChIP: Chromatin immunoprecipitation
CREB: cAMP-response element-binding protein
cAMP: Cyclic adenosine monophosphate
CBP: CREB binding protein
DAI: Disease activity index
DSS: Dextran sulfate sodium
DIP: Dipyridamole
GPX4: Glutathione peroxidase 4
GSH: Glutathione
IP: Immunoprecipitation
IBD: Inflammatory bowel disease
IF: Immunofluorescence
IECs: Intestinal epithelial cells
MUC2: Mucin2
MAC: Membrane attack complex
NLRP3: NLR family, pyrin domain containing 3
NF-*κ*B: Nuclear factor *κ*B
PDE4: Phosphodiesterase 4
PKA: Protein kinase A
Rofi: Roflumilast
ROS: Reactive oxygen species
SES-CD: Simple endoscopic score for Crohn's disease
SLC7A11: Solute carrier family 7 member 11
TNF-*α*: Tumor necrosis factor-*α*
UC: Ulcerative colitis
VTN: Vitronectin
WB: Western blotting
ZO-1: Tight junction protein-1.

## Supplementary Materials

Supplementary Materials Figure S1: VTN enhanced cellular ROS level to induced ferroptosis (the detailed information of subjects enrolled in the study).
