# Supplementary material for: Vitronectin Destroyed Intestinal Epithelial Cell Differentiation through Activation of PDE4-Mediated Ferroptosis in Inflammatory Bowel Disease
Source: Mediators Inflamm. 2023 Jul 19;2023:6623329. doi: 10.1155/2023/6623329 (PMC10371469; doi:10.1155/2023/6623329)
Supplement: Supplementary Materials — Figure S1: VTN enhanced cellular ROS level to induced ferroptosis (the detailed information of subjects enrolled in the study). [file 6623329.f1.docx]

**Supplementary Materials**

**Figure legend**

**Fig.S1 VTN enhanced cellular ROS level to induced ferroptosis.** (A-B) HT-29 cells were digested and reseeded into 6-well plate overnight, after serum starvation for 12 hours, cells were stimulated as indicated for further 48 hours, and the level of cellular ROS was determined according to the manufactory instruction and captured under microscopy. **(C)** WB was used to verify the knockdown efficiency of PDE4s in HT-29 cells transfected with indicated siRNAs.

**
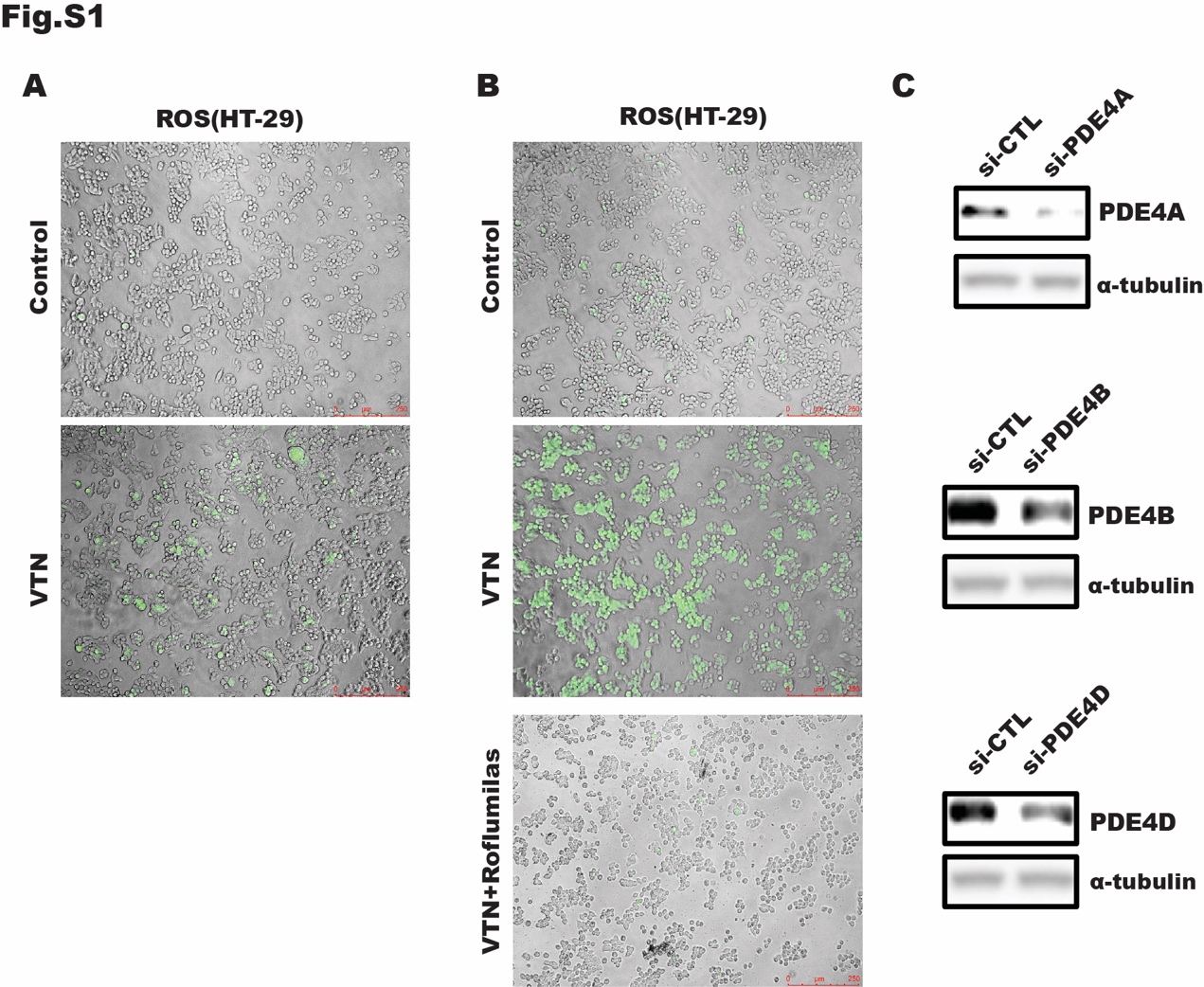
**
